# Rendering Mortars Reinforced with Natural Sheep’s Wool Fibers

**DOI:** 10.3390/ma12223648

**Published:** 2019-11-06

**Authors:** Cinthia Maia Pederneiras, Rosário Veiga, Jorge de Brito

**Affiliations:** 1 CERIS, Instituto Superior Técnico, University of Lisbon, 1049-001 Lisbon, Portugal; cinthiamaia@tecnico.ulisboa.pt; 2 National Laboratory for Civil Engineering, 1700-066 Lisbon, Portugal; rveiga@lnec.pt

**Keywords:** render, cement and cement-lime reinforced mortars, natural fiber, sheep’s wool, sustainability

## Abstract

The susceptibility of rendering mortars to cracking is a complex phenomenon. Fibers have been incorporated in mortars to ensure the durability of the render and can improve the flexural strength, fracture toughness, and impact resistance of the mortars. Aside from the better cracking performance of fiber reinforced mortars, natural fibers have been a path to reducing the environmental impacts of construction materials. Recycling has high sustainability-related potential as it can both mitigate the amount of waste being inadequately disposed and reduce the consumption of natural raw materials. Studies on the incorporation of waste in civil engineering materials have been growing, and recycled fibers may be feasible to incorporate in mortars. Natural fibers are considered as a viable replacement for synthetic ones. Several studies have investigated vegetal fibers in cementitious composites. However, only a few have focused on the incorporation of waste animal-based fiber. The aim of this work is to analyze the feasibility of the use of natural sheep’s wool fibers on the reinforcement of mortars and in particular to improve their cracking behavior. For this purpose, two different binders were used: cement and cement-lime mortars were produced. The incorporation of 10% and 20% (in volume) of 1.5 cm and 3.0 cm wool fibers was analyzed. The results show that the incorporation of wool fibers increased the ductility of the mortars and improved their mechanical properties.

## 1. Introduction

The construction sector has been trying to reduce its environmental impacts. Eco-friendly constituents have been an alternative to develop new cementitious materials. In order to enhance the cracking performance of mortars, the incorporation of natural fibers can contribute to better performance while ensuring a sustainable approach. According to previous studies, natural fibers such as sheep wool may improve the ductility of cementitious composites and also provide an adequate disposal of the waste [1].

The incorporation of fibers enhances a better post-cracking behavior due to the higher fracture toughness, flexural strength, and impact resistance of the mortars [2]. The benefits of fiber reinforcement in cementitious materials depend on the fiber type, their geometry, and their volume ratio and distribution [3]. The use of natural fibers compared to man-made fibers has been achieving environmental, energy, and resource conservation benefits [4]. There are three types of natural fibers: plant-based, mineral-derived, and animal-based. In this paper, the study focused on animal-based fibers, namely sheep’s wool fibers.

Natural sheep wool is considered as waste on a large-scale, taking into account that 270.000 tons of wool are produced by 90 million sheep in Europe [3]. Indeed, 75% of the wool produced (around 150 million tons per year) is rejected by the textile industry [4,5]. Most of this material does not have a proper disposal method. Furthermore, the wool fibers have an elastic modulus of about 1–4 GPa, which can be comparable to the modulus of plastic fibers [5].

Several studies have been carried out to investigate the incorporation of wool fibers in cementitious materials to improve the thermal insulation properties [6,7,8,9]. However, only a few studies, described next, have incorporated wool fibers with the purpose of improving the mechanical performance of cementitious composites.

Alyousef et al. [10] analyzed the mechanical properties of reinforced concrete with wool fibers and found that the incorporation of fibers decreased the workability of concrete. Concerning the mechanical behavior, the fibers enhanced the ductility and flexural capacity of the composite. In terms of compressive strength, the incorporation of fibers implied a reduction of strength. This reduction was explained by the authors by the incorporation of wool up to 6% by weight of cement in the concrete, which led to a reduction of the total binder in the mix.

Fantilli et al. [5] investigated the incorporation of 1% (in volume) of wool fibers in cementitious reinforced-mortars. The authors observed that the fibers reduced the brittle nature of the mortars due to the development of bridge mechanisms between the crack borders. In this research, an improvement in the fracture toughness in the reinforced mortars of 300% relative to the control mortar is reported. It could also be seen that the wool fibers promoted a reduction in the plastic shrinkage. The cementitious mortars reinforced with wool fibers presented the same mechanical performance (i.e., strengths and modulus of elasticity) as those in mortars with the most common vegetal fibers.

Giosué et al. [11] investigated the replacement of 25% (in volume) of aggregates with wool fibers in lime-based lightweight mortars. The modified mortars were tested in the fresh and hardened state concerning workability, mechanical strength, and hygro-thermal properties. The results showed an increase of about 30% in the flexural strength of the modified mortars.

Kesikidou and Stefanidou [12] investigated the incorporation of natural fibers in mortars. The authors analyzed mortars with two different binders, incorporating vegetal fibers such as jute, coconut, and kelp and showed that the natural fibers performed differently in relation to cement or lime-based binder. Lime-reinforced mortars presented a higher increase in flexural and compressive strength when compared to the cement-based reinforced mortars. Therefore, the compatibility of the fibers with the mortar’s composition should be evaluated.

As a conclusion of this review, it was found that, aside from all of the studies carried out on mortars with natural fibers, the incorporation of wool fibers in rendering cementitious mortars could not be found in the technical literature. This is relevant because rendering mortars mainly have the function of protecting the substrate. This means that a high compressive strength is not often necessary, but is important to minimize cracking. As a consequence, usually low strength mortars with a low binder/aggregate ratio are used, and from this aspect, leads to a different microstructure and a poorer adhesion between the binder, aggregates, and fibers.

Additionally, the compositions used result in a low modulus of elasticity, possibly nearer the modulus of elasticity of natural fibers than in the case of structural mortars. Finally, for rendering mortars, as a favorable cracking behavior is more important than a high mechanical strength, characteristics such as a low modulus of elasticity and ductility are mainly required. Therefore, the novelty of this work was in analyzing the feasibility of the incorporation of wool fibers in rendering mortars and their efficacy in improving those specific properties. Cement and cement-lime mortars with low binder/aggregate volumetric ratios of 1:4 and 1:3 were produced, respectively, with different fibers lengths of 1.5 and 3.0 cm, respectively. The ratios of incorporation were 10% and 20% of the total mortar volume.

The mortars’ properties considered relevant for rendering mortars, namely workability, bulk density, dynamic modulus of elasticity, ultra-sound pulse velocity, flexural and compressive strengths, and protection to water action, were evaluated in the fresh and hardened states.

From the results, it could be seen that the incorporation of wool fibers in rendering mortars with a volumetric ratio of 1:4 (cement: aggregates) and 1:1:6 (cement:air-lime:aggregates) improved the cracking behavior of the materials as the modified mortars, in general, presented a higher flexural strength and a lower modulus of elasticity when compared to the plain mortars.

## 2. Experimental Program

The aim of this research was to evaluate the influence of natural fiber waste, namely sheep wool, incorporated in rendering mortars.

Cement mortars and cement-air lime-based mortars were produced. The volumetric proportions were 1:4 (cement: aggregates) and 1:1:6 (cement:air-lime:aggregates). Aside from the binder, the length and volume ratio were parameters used to formulate the analyzed mortars.

Mortars were identified as follows:

- REF 1:4 (0% of incorporation—reference cement mortar)
- W 1.5_10%c (10% of incorporation of 1.5 cm long wool fibers—cement mortar)
- W 3.0_10%c (10% of incorporation of 3.0 cm long wool fibers—cement mortar)
- W 1.5_20%c (20% of incorporation of 1.5 cm long wool fibers—cement mortar)
- W 3.0_20%c (20% of incorporation of 3.0 cm long wool fibers—cement mortar)
- REF 1:1:6 (0% of incorporation—reference cement-lime mortar)
- W 1.5_10%cl (10% of incorporation of 1.5 cm long wool fibers—cement-lime mortar)
- W 3.0_10%cl (10% of incorporation of 3.0 cm long wool fibers—cement-lime mortar)
- W 1.5_20%cl (20% of incorporation of 1.5 cm long wool fibers—cement-lime mortar)
- W 3.0_20%cl (20% of incorporation of 3.0 cm long wool fibers—cement-lime mortar)

### 2.1. Materials

**Figure 1:**
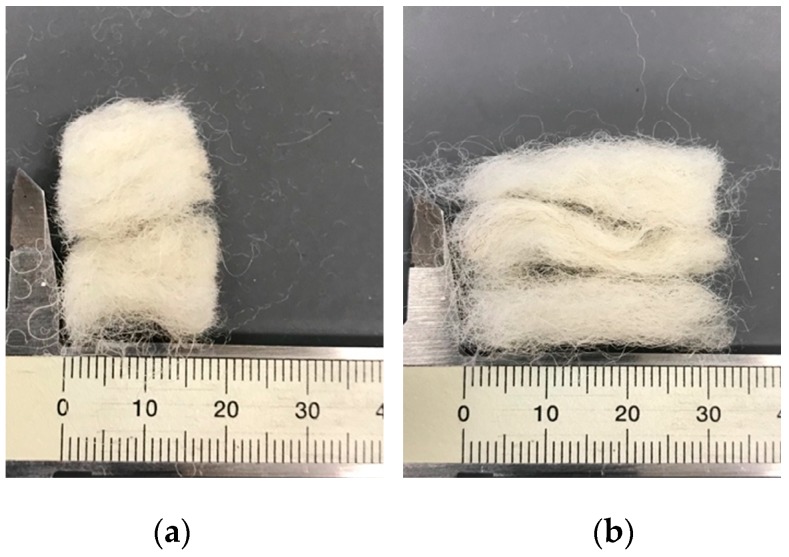
Sheep wool fiber used: (**a**) 1.5 cm long (**b**) 3.0 cm long.

The materials used were cement, air-lime, sand, and wool fibers. The binder used on the cement mortars was CEM II/B-L 32.5 N, according to EN 197-1 [13]. The calcium hydrated lime powder—air lime—used was class CL80-S, according to EN 459-1 [14]. The natural silica sand was previously washed, calibrated, and sieved for a required size distribution. The wool fibers were washed with neutral detergent, and dried at 40 °C. This procedure was applied to remove the impurities. The fiber length was obtained by manually cutting the waste material. The fibers were added in order to ensure a homogeneous dispersion in each mix composition. The homogeneity of the fibers in the mix was implemented by distributing them in a properly closed receptacle and blowing compressed air over them in order to achieve an adequate dispersion before adding to the mix. Figure 1 presents the wool fibers used in the experimental campaign. The apparent bulk density of the constituents of the mortars produced is presented in Table 1.

Table 2 presents the composition of the mortars produced in this work.

### 2.2. Methods

All tests carried out in the experimental program are described in Table 3.

For the hardened mortar tests, prismatic samples (40 mm × 40 mm × 160 mm) were used, in accordance with European Standards.

For ultra-sound pulse velocity, the direct and indirect methods were used for the measurements. In the direct transmission method, the electrodes are placed on the opposite surfaces of the specimen. In the indirect method, the electrodes are positioned on the same surface of the prism: the transmitter electrode is fixed at a specific point and the receptor moves over the specimen, and at different distances, the transmission time is measured allowing for the velocity to be calculated.

In order to analyze the susceptibility to cracking of the mortars produced in this work, some parameters were calculated. The Center Scientifique et Technique du Bâtiment (CSTB) [15] refers to the dynamic modulus of elasticity and flexural strength ratio (E/σ_f_) as indicators of the mortar’s ability to resist cracking. This criterion is based on the fact that a lower dynamic modulus of elasticity provides a higher deformation capacity of the material, and a greater flexural strength induces the material to withstand tensions without cracking. Therefore, the tendency to crack due to restrained shrinkage is greater when the ratio between the modulus of elasticity and tensile strength is high.

Another parameter to evaluate the susceptibility to crack is based on the flexural and compressive strengths. The ductility of the material can be associated to this ratio (σ_f_/σ_c_) (i.e., the mortar is considered more ductile when this value is closer to 1). Ductility is a measure of the deformability of the material before fracture. Cracking resistance is correlated with the deformation capacity of the mortar and its ability to absorb stress without cracking [23].

The ability to absorb energy before fracture is correlated with the toughness of the mortar. The fracture toughness was calculated by the total area under the strain–stress curve of the results of flexural strength at 28 days.

Regardless of the binder used, all hardened mortars were cured as specified by EN 1015-11 [21]. The specimens were kept in molds for two days at a temperature of 20 ± 2 °C and a relative humidity of 95 ± 5%. After demolding, all specimens were maintained in the same conditions for a total of seven days. After that, the specimens were kept at 20 ± 2 °C and the relative humidity was reduced to 65 ± 5%, until testing.

## 3. Results and Analysis

### 3.1. Fresh State

**Figure 2:**
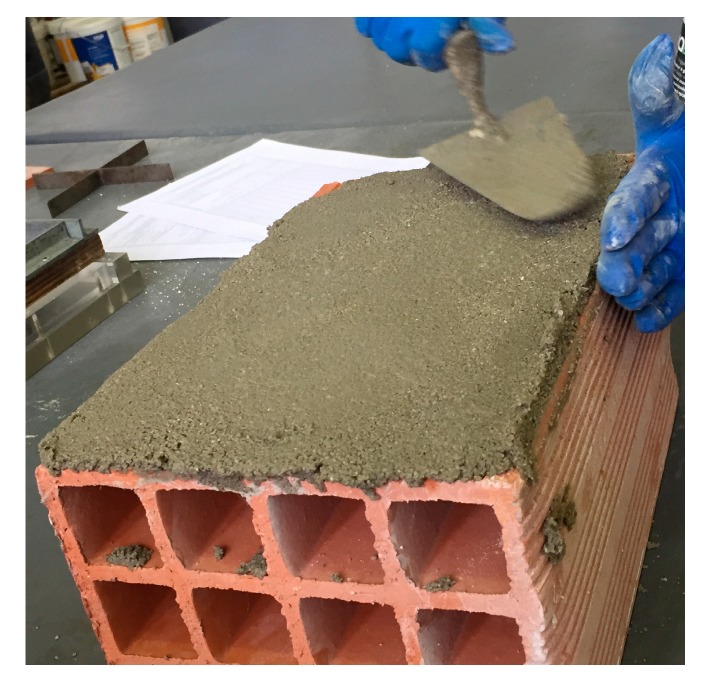
Mortar application on a brick.

The mortar’s workability was measured by the consistency test. To improve the comparability of the results, the values were limited to 140 ± 5 mm. The mortars presented a stiff consistence, but an application on a brick was carried out to ensure that an adequate workability was achieved, as shown in Figure 2.

**Figure 3:**
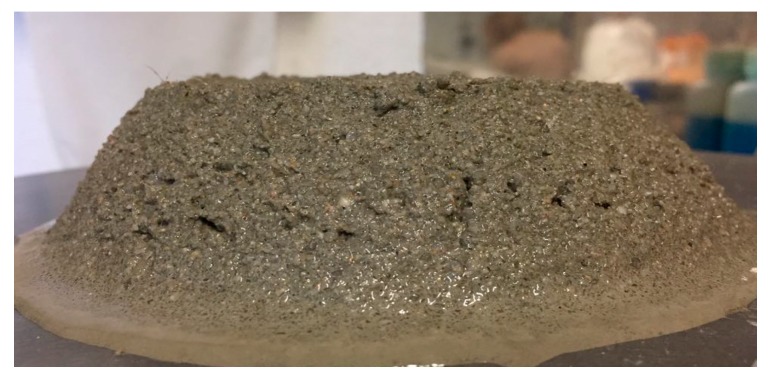
Flow table test for the modified mortar.

It was noticed that the fibers kept the mortar agglutinated. Although the workability was acceptable, the flor value did not increase due to the fibers’ agglutinating action. In order to illustrate this behavior, Figure 3 presents the flow table test of the W 1.5_10%c sample.

Table 4 presents the results of the fresh mortars’ properties.

In all cement-lime mortars, the water/binder ratio was higher than that of the cement mortars. The incorporation of 10% of wool fibers increased the amount of water needed to maintain the workability. This could be due to the morphology of the fiber, which is composed of keratin filaments [5]. However, the incorporation of 20% of fibers again decreased the water/binder ratio to values similar to the control mortar. This trend reversion may be due to the fact that the longer fibers have a lower bulk density and thus a lower weight of incorporated fibers is actually used (Table 2). The modified mortars presented a lower bulk density than that of the reference mortars.

### 3.2. Hardened State

#### 3.2.1. Dry Bulk Density of the Hardened Mortars

**Figure 4:**
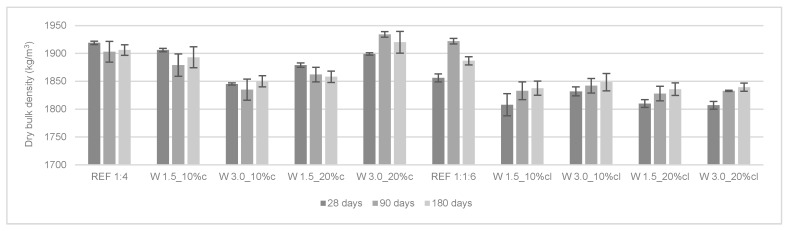
Dry bulk density of the hardened mortars.

The dry bulk density of the hardened mortars was determined at 28, 90, and 180 days and the results are presented in Figure 4. The same trend as for the fresh state was noticed: the incorporation of wool fibers reduced the dry bulk density of the mortars due to the low bulk density of the fibers. Giosué et al. [11] found similar results (i.e., the bulk density of the reinforced mortars had a decrease of about 13% compared to the control mortar at 28 days).

In general, the dry bulk density of the cement mortars decreased from 28 to 90 days. Dry bulk density is defined as the ratio between mass and volume. Thus, the variations in weight and volume during time explain the variations in dry bulk density. In a previous study, a similar trend of decrease from 28 to 90 days was found [24], possibly because the mass reduction overlapped the volume reduction.

The opposite effect occurred in the cement-lime mortars, which presented an increase in this property over time. In fact, cement-lime mortars also have shrinkage with the consequent reduction of volume, and possibly have an increase in weight due to a higher carbonation reaction (by comparison with cement-only mortars). Previous works have shown this same trend for air lime mortars [25].

#### 3.2.2. Dynamic Modulus of Elasticity of the Hardened Mortars

**Figure 5:**
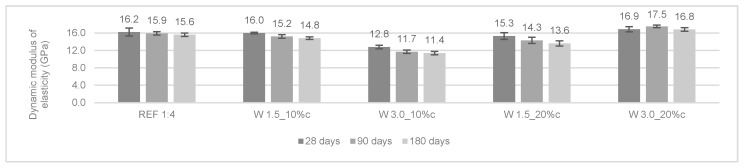
Dynamic modulus of elasticity of the cement mortars.

**Figure 6:**
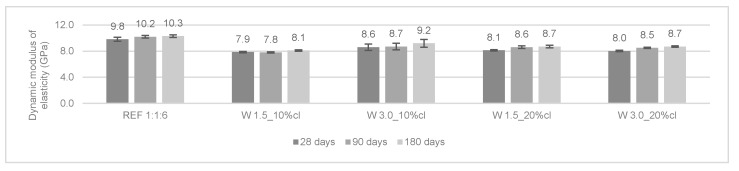
Dynamic modulus of elasticity of the cement-lime mortars.

The modulus of elasticity measures the ability of the rendering mortars to absorb deformations. The renders should be able to withstand higher internal stresses without cracking. The dynamic modulus of elasticity was determined at 28, 90, and 180 days. The results are presented in Figure 5 and Figure 6.

In general, the modified mortars presented a lower modulus of elasticity when compared to the reference mortars. For cement mortars, the modulus of elasticity decreased from 28 to 180 days. This could be attributed to the internal micro-cracking of the mortars over time. The opposite trend occurred in cement-lime mortars (i.e., the modulus of elasticity presented a slight increase from 28 to 90 days).

According to Araya-Letelier et al. [26], the incorporation of pig hair in mortars did not lead to a significant reduction in the dynamic modulus of elasticity. The authors explained this effect by the small amount of total fiber volume incorporated (up to 1.5%).

#### 3.2.3. Ultra-Sound Pulse Velocity of the Hardened Mortars

The ultra-sound pulse velocity test was performed at 28, 90, and 180 days. The results are presented in Table 5. The ultra-sound pulse velocity results showed that the incorporation of fibers reduced the pulse velocity through the mortar, indicating a decrease in the modulus of elasticity (Table 5).

**Figure 7:**
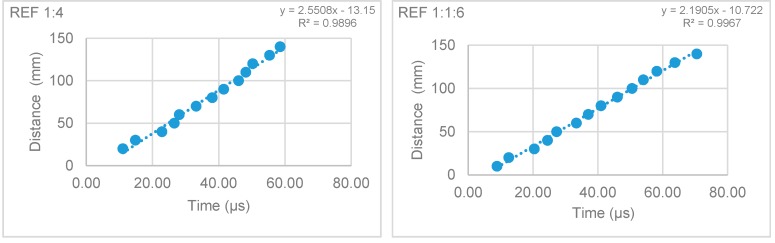
Ultra-sound pulse velocity of the reference mortars.

**Figure 8:**
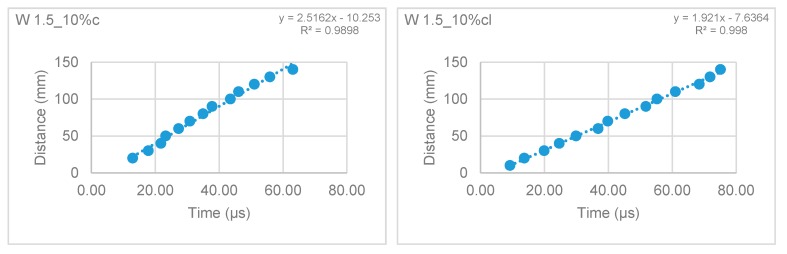
Ultra-sound pulse velocity of the W 1.5_10% mortar.

These results followed the same trend as those of the modulus of elasticity test, as expected. The direct method showed a decrease of the pulse velocity in cement mortars, which could be related to some internal cracks due to shrinkage. According to the indirect method, this reduction is not that significant as it measures the velocity in small distances in the prism, which may detect a more distributed crack pattern and consequent decrease of the pulse velocity, as seen by the high R^2^ of the velocity trend lines (Figure 7 and Figure 8).

Figure 5 and Figure 6 present the results of the ultra-sound pulse velocity test by the indirect method for the reference mortars and W 1.5_10% mortars.

#### 3.2.4. Flexural and Compressive Strength of the Hardened Mortars

**Figure 9:**
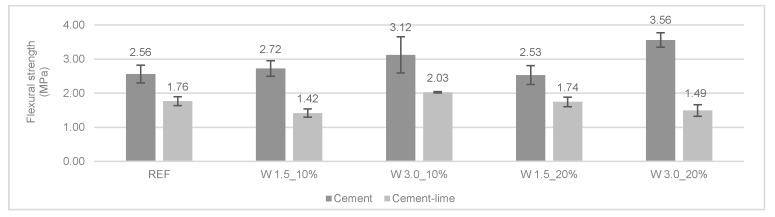
Flexural strength of the mortars at 28 days.

**Figure 10:**
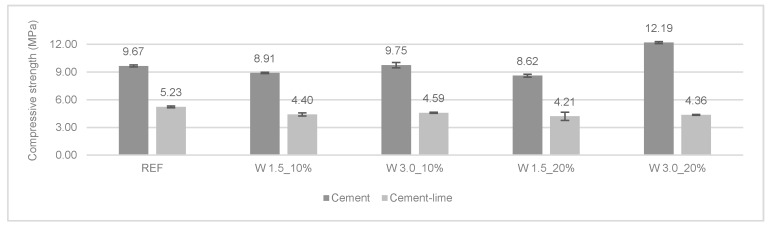
Compressive strength of the mortars at 28 days.

Figure 9 and Figure 10 present the results of the flexural and compressive strengths at 28 days. In general, the incorporation of wool fibers in cement mortars improved their flexural strength. Longer fibers (3.0 cm) presented a higher increase in flexural strength. The cement mortar with 20% of 3.0 cm long wool fibers had an increase of 40% and 26% in flexural and compressive strength, respectively, compared to REF 1:4.

**Figure 11:**
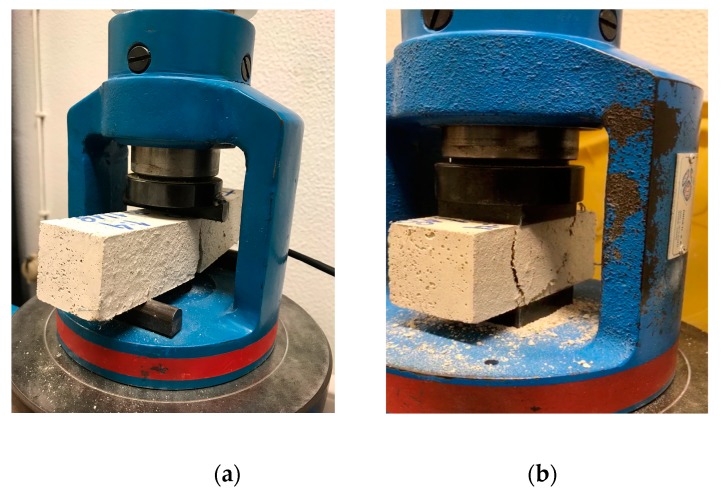
Flexural strength test (**a**); compressive strength test (**b**).

For cement-lime mortars, only W 3.0 cm 10% had an increase of 15% in this property when compared to REF 1:1:6. In general, the modified cement-lime mortars presented a slight decrease in flexural strength. W 1.5 cm 10% obtained the lowest flexural strength, 20% less than the reference mortar. Figure 11 presents the mechanical tests carried out on the mortars.

Fantilli et al. [5] reached similar conclusions to those of the cement mortars. The modified mortars with wool fibers had an 18% higher flexural strength than that of the control mortar. Araya-Letelier et al. [23] studied the incorporation of natural animal fibers such as pig hair in mortars and also reported an increase in flexural strength of the modified mortars. Giosué et al. [11] reported that a hydraulic-lime mortar with wool fibers achieved about a 30% higher flexural strength than that of the conventional mortar. These previous works explained this increase in flexural strength due to a bridging mechanism.

In the compressive strength test, a different trend was found regarding the type of the binder used and the length of the fibers. Indeed, it can be seen that the longer fibers led to an increase in the compressive strength when compared to the cement mortar reference. Modified cement mortars with 1.5 cm of wool fiber presented a slight decrease in compressive strength.

The modified cement-lime mortars obtained a slight reduction of compressive strength compared to the control mortar (REF 1:1:6). W 1.5_20% cl obtained the most significant decrease compared to the reference mortar, about 20%.

Giosué et al. [11] found a decrease in the modified lime-based mortars with wool fibers. The authors reported that the use of fibers reduced the compressive strength of mortars. In agreement with this study, Araya-Letelier et al. [26] found a reduction of compressive strength with the incorporation of animal-based fibers in cement mortars.

**Figure 12:**
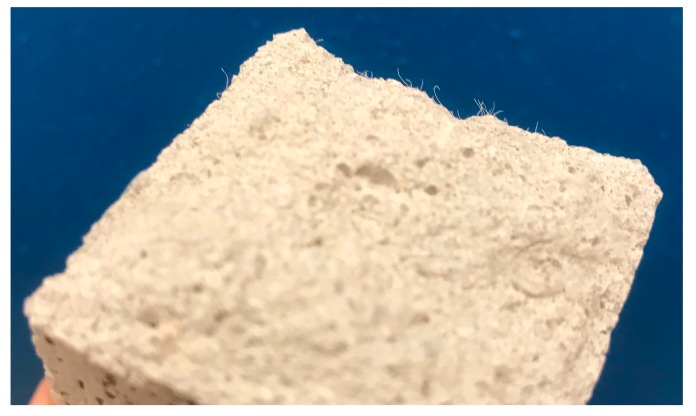
Sample of the modified mortar with wool fiber incorporation.

Figure 12 presents a sample of the modified mortar, where the wool fibers do not produce a significant change in the mortar’s appearance.

#### 3.2.5. Cracking Behavior

Restrained shrinkage can be induced by the restrictions imposed on deformations of a rendering mortar. Tensile stresses should be dissipated without cracking of the coating. There are several causes that lead the mortar to crack. In order to evaluate the mortar’s susceptibility to cracking, some parameters were considered to enhance the analysis of this phenomena as described above, and the results are presented in Table 6.

The values of fracture toughness are presented in Table 6.

An increase in the fracture toughness of the modified cement mortars was noticed. The increment was higher when longer fibers were incorporated, regardless of the incorporation ratio. W 3.0_10%c and W 3.0_20%c attained up to 100% higher toughness values when compared with the reference cement mortar (REF 1:4).

The fracture toughness results of the cement-lime mortars did not present significant changes.

These results are in accordance with previous studies [5,27]. Fantilli et al. [5] also obtained a higher fracture toughness with the incorporation of wool fibers in cementitious composites. This can be explained by the fibers’ bridging mechanism, since the fibers cross the micro-cracks, preventing their propagation and delaying the occurrence of the first crack. Reinforced mortars may withstand tensile load after cracking and exhibit ductile behavior [1,28].

Araya-Letelier et al. [26] found that the incorporation of animal-based fibers, namely pig hair, increased the fracture toughness of the fiber-reinforced mortars. The authors related this increment to the increase in the impact energy absorption capacity of the mortars due to the addition of fibers. In this work, it was reported that the post-cracking behavior of the modified mortars was improved by up to 55% higher energy absorbed at failure.

Considering these factors, all the modified mortars evaluated in this work were less susceptible to cracking. However, the cement-lime mortars presented more ductility than the cement mortars. The incorporation of 20% of 1.5 cm long wool fibers in cement-lime mortars presented the best results regarding ductility. Table 6 presents the results of tests on the hardened mortars.

#### 3.2.6. Open Porosity

The open porosity test determines the volume of interconnected voids in the mortars, in percentage. This property is correlated with the ultra-sound pulse velocity and modulus of elasticity as well as the mechanical strength and water tightness behavior. Table 7 presents the results of the open porosity test.

The results of the open porosity test confirmed the expectations. In general, the incorporation of fibers increased the volume of pores of the modified mortars. W 3.0_20%c was an exception, as it presented a reduction of 1.5% of total open porosity compared to the control cement mortar. The incorporation of 10% of wool fibers in cement mortars presented the same values, regardless of the length of the fibers.

The cement-lime modified mortars exhibited a greater increase in the volume of pores than that of the cement mortars. W 3.0_20% cl obtained an increase of about 6.5% of total open porosity compared to REF 1:1:6.

Giosué et al. [11] also noticed an increase (32%) of total open porosity in hydraulic lime-mortars with the incorporation of 25% of wool fibers when compared to the control mortar. The increase in open porosity of the modified mortars could be explained by the fiber–matrix interfacial bond that is thought to be less efficient than that of the sand–matrix.

## 4. Conclusions

From the results of the experimental campaign, it was concluded that the incorporation of wool fibers in rendering mortars presented a satisfactory performance concerning the mechanical and cracking behavior. All of the modified mortars presented less susceptibility to cracking when compared with the mortars without fibers, based on the parameters evaluated.

In general, the reinforced mortars presented a decrease in the dynamic modulus of elasticity, which can be considered an advantage of the incorporation of wool fibers. W 3.0_10%c and W 1.5_10%cl obtained a similar decrease of 20% of modulus of elasticity when compared to the control mortar.

Regarding flexural strength, the modified cement mortars presented an increase when compared to the reference mortar. The addition of longer fibers enhanced the mechanical strength of the mortars. W 3.0_20%c obtained an increase of 40% in flexural strength when compared to REF 1:4. The cement-lime modified mortars obtained a slight reduction in flexural strength, with the exception of W 3.0_10%cl.

The dynamic modulus of elasticity and flexural strength ratio (E/σ_f_) was analyzed as an indicator of the mortars’ ability to resist cracking. It could be seen that all of the modified mortars, regardless of the binder used, presented a lower (E/σ_f_) ratio, which could lead to a lesser tendency to crack due to restrained shrinkage when compared to the reference mortars.

The ratio (σ_f_/σ_c_) is related to the mortar’s ductility. According to the results, all the modified mortars presented a higher ratio (σ_f_/σ_c_) compared to the reference mortars, which allows concluding that the incorporation of fibers increased the ductility of those mortars, based on this parameter.

Regarding fracture toughness, the modified cement mortars presented improvements. In fact, the toughness increment was higher when longer fibers were incorporated. However, for the cement-lime mortars, the toughness results did not present any significant contribution of the incorporation of the fibers.

Besides the analyzed parameters related with the cracking behavior, it was also found that, concerning compressive strength, the incorporation of longer fibers in cement mortars (W 3.0_10%c and W 3.0_20%c) resulted in an increase.

The results obtained in this work identify the advantages of the addition of the natural wool fibers in rendering mortars, namely concerning the improvement in the mechanical, and in particular of the cracking behavior.

## Figures and Tables

**Table 1:** Apparent bulk density of the constituents.

Component	Apparent Bulk Density (kg/m^3^)
Cement	975.5
Air-lime	565.7
Sand	1230.8
Wool 1.5 cm	4.25
Wool 3.0 cm	2.53

**Table 2:** Composition of the mortar mixes by mass.

Mortar	Cement (g)	Air-lime (g)	Sand (g)	Water (g)	Fibers (g)
REF 1:4	487.8	-	2461.6	445	-
W 1.5_10%c	439.1	-	2215.4	405	1.1
W 3.0_10%c	439.1	-	2215.4	435	0.6
W 1.5_20%c	390.2	-	1969.3	350	2.1
W 3.0_20%c	390.2	-	1969.3	350	1.3
REF 1:1:6	304.8	176.8	2307.8	465	-
W 1.5_10%cl	274.4	159.1	2077.0	435	1.1
W 3.0_10%cl	274.4	159.1	2077.0	420	0.6
W 1.5_20%cl	243.9	141.4	1846.2	370	2.1
W 3.0_20%cl	243.9	141.4	1846.2	370	1.3

**Table 3:** Experimental campaign tests.

Test	European Standard	Samples	Specimens	Age (days)
Apparent bulk density	Cahier 2669-4 [15]	6	Cement, lime, sand and fibers	-
Consistence by flow table	EN 1015-3 [16]	3	Fresh mortar	-
Bulk density	EN 1015-6 [17]	3	Fresh mortar	-
Dry bulk density	EN 1015-10 [18]	3	Hardened mortar	28, 90, and 180
Dynamic modulus of elasticity by resonance frequency method	EN 14146 [19]	3	Hardened mortar	28, 90. and 180
Ultra-sound pulse velocity	EN 12504-4 [20]	1	Hardened mortar	28, 90, and 180
Flexural and compressive strengths	EN 1015-11 [21]	3	Hardened mortar	28
Open porosity	EN 1936 [22]	3	Hardened mortar	28

**Table 4:** Fresh mortar properties.

Mortar	w/b Ratio	Consistency (mm)	Bulk Density (kg/m^3^)
REF 1:4	0.91	140	2005.3
W 1.5_10%c	0.92	141	1978.1
W 3.0_10%c	0.98	139	1969.5
W 1.5_20%c	0.89	135	1936.5
W 3.0_20%c	0.89	141	2002.9
REF 1:1:6	0.98	140	1998.8
W 1.5_10%cl	1.02	141	1980.3
W 3.0_10%cl	0.98	141	1977.8
W 1.5_20%cl	0.97	139	1970.3
W 3.0_20%cl	0.97	139	1985.9

**Table 5:** Ultra-sound pulse velocity of the mortars tested.

Mortar	Ultra-Sound Pulse Velocity (m/s)					
	Direct Method			Indirect Method		
	28 days	90 days	180 days	28 days	90 days	180 days
REF 1:4	2855	2751	2661	2551	2581	2676
W 1.5_10%c	2727	2695	2650	2516	2530	2361
W 3.0_10%c	2641	2493	1842	2830	2293	2399
W 1.5_20%c	2556	2602	1855	2677	2739	2545
W 3.0_20%c	2788	2925	1892	2652	2779	2731
REF 1:1:6	2205	2218	2321	2190	2059	2321
W 1.5_10%cl	2050	1990	2051	1921	1968	2003
W 3.0_10%cl	2118	2169	2219	2039	2108	2050
W 1.5_20%cl	2040	2076	2143	2136	2071	2276
W 3.0_20%cl	1985	2065	2120	1957	1936	2017

**Table 6:** The mechanical test results of the hardened mortars and parameters related to cracking.

Mortar	Dry Bulk Density (kg/m^3^)	Dynamic Modulus of Elasticity (MPa)	Flexural Strength (MPa)	Compressive Strength (MPa)	E/σ_f_	σ_f_/σ_c_	Fracture Toughness (N.mm)
REF 1:4	1919	16210 ± 0.91	2.56 ± 0.21	9.66 ± 0.11	6332	0.27	0.195 ± 0.07
W 1.5_10%c	1906	15980 ± 0.19	2.72 ± 0.19	8.75 ± 0.08	5875	0.31	0.278 ± 0.04
W 3.0_10%c	1845	12790 ± 0.29	3.12 ± 0.43	9.47 ± 0.25	4099	0.33	0.415 ± 0.05
W 1.5_20%c	1879	15290 ± 0.61	2.53 ± 0.22	8.53 ± 0.16	6043	0.30	0.235 ± 0.06
W 3.0_20%c	1899	16860 ± 0.47	3.56 ± 0.17	11.50 ± 0.11	4736	0.31	0.392 ± 0.06
REF 1:1:6	1856	9820 ± 0.21	1.76 ± 0.10	5.22 ± 0.09	5580	0.34	0.135 ± 0.02
W 1.5_10%cl	1808	7850 ± 0.09	1.42 ± 0.09	3.28 ± 0.15	5528	0.43	0.090 ± 0.01
W 3.0_10%cl	1832	8600 ± 0.41	2.03 ± 0.01	4.48 ± 0.06	4236	0.45	0.147 ± 0.03
W 1.5_20%cl	1810	8130 ± 0.05	1.74 ± 0.11	3.17 ± 0.40	4672	0.55	0.127 ± 0.02
W 3.0_20%cl	1807	8030 ± 0.04	1.49 ± 0.13	2.99 ± 0.05	5389	0.50	0.104 ± 0.02

**Table 7:** Open porosity test results of the hardened mortars.

Mortar	Open Porosity (%)
REF 1:4	20.18 ± 0.003
W 1.5_10%c	20.91 ± 0.001
W 3.0_10%c	20.91 ± 0.007
W 1.5_20%c	20.82 ± 0.003
W 3.0_20%c	19.88 ± 0.003
REF 1:1:6	23.93 ± 0.003
W 1.5_10%cl	24.93 ± 0.008
W 3.0_10%cl	24.65 ± 0.003
W 1.5_20%cl	24.73 ± 0.008
W 3.0_20%cl	25.49 ± 0.012
